# Research Advances in Cardio-Cerebrovascular Diseases of *Ligusticum chuanxiong* Hort.

**DOI:** 10.3389/fphar.2021.832673

**Published:** 2022-01-31

**Authors:** Dan Li, Yu Long, Shuang Yu, Ai Shi, Jinyan Wan, Jing Wen, Xiaoqiu Li, Songyu Liu, Yulu Zhang, Nan Li, Chuan Zheng, Ming Yang, Lin Shen

**Affiliations:** ^1^ State Key Laboratory of Southwestern Chinese Medicine Resources, Chengdu University of Traditional Chinese Medicine, Chengdu, China; ^2^ Key Laboratory of Modern Preparation of Traditional Chinese Medicine, Ministry of Education, Jiangxi University of Traditional Chinese Medicine, Nanchang, China; ^3^ Second Affiliated Hospital of Tianjin University of Traditional Chinese Medicine, Tianjin, China

**Keywords:** *Ligusticum chuanxiong* Hort., cardio-cerebrovascular diseases, material basis, molecular mechanism, clinical efficacy

## Abstract

Cardio-cerebrovascular diseases (CVDs) are a serious threat to human health and account for 31% of global mortality. *Ligusticum chuanxiong* Hort. (CX) is derived from umbellifer plants. Its rhizome, leaves, and fibrous roots are similar in composition but have different contents. It has been used in Japanese, Korean, and other traditional medicine for over 2000 years. Currently, it is mostly cultivated and has high safety and low side effects. Due to the lack of a systematic summary of the efficacy of CX in the treatment of CVDs, this article describes the material basis, molecular mechanism, and clinical efficacy of CX, as well as its combined application in the treatment of CVDs, and has been summarized from the perspective of safety. In particular, the pharmacological effect of CX in the treatment of CVDs is highlighted from the point of view of its mechanism, and the complex mechanism network has been determined to improve the understanding of CX’s multi-link and multi-target therapeutic effects, including anti-inflammatory, antioxidant, and endothelial cells. This article offers a new and modern perspective on the impact of CX on CVDs.

## 1 Introduction

Cardio-cerebrovascular diseases (CVDs) are the general name of cardiovascular and cerebrovascular diseases (Liu et al., 2021). They generally refer to the ischemic or hemorrhagic diseases of the heart, brain, and the whole body caused by hyperlipidemia, blood viscosity, atherosclerosis (AS), hypertension, etc. These include coronary heart disease (CHD), myocardial infarction, angina pectoris, coronary artery insufficiency, aortic AS, and other cardiovascular diseases, as well as ischemic stroke, hemorrhagic stroke, transient cerebral ischemia and other cerebrovascular diseases (Qin, 2020). CVDs can occur alone, but often exist at the same time in different degrees. CVDs is a common and serious threat to human health, with the characteristics of high morbidity, high disability rate, high recurrence rate, and high mortality. More than 31% of deaths worldwide are attributable to cardiovascular diseases, and more than any the other causes of death, this is the main cause of incidence rate and mortality rate in developing and developed countries (Stephanieh and Sarahh, 2020). The burden of global CVDs is increasing day by day, which has become a major public health problem. At present, there are more than 290 million patients with CVDs in China, accounting for 42.5 and 44.6% of the deaths in urban and rural areas (Zhou et al., 2019a). More than 80% of the cardiovascular disease burden could be attributed to known modifiable risk factors such as hypertension, dietary risks, high low-density lipoprotein cholesterol, and impaired kidney function (Paoloa et al., 2020). Hypertension, high cholesterol, and diabetes are the three major risk factors for CVDs, among which hypertension is the primary factor (Wang, 2016).

As we enter a golden age of drug discovery based on natural products, the growing interest in natural products has led to the discovery of new chemical entities for the treatment of various human diseases in the past decades. Natural drugs extracted from herbal medicines are valuable sources of chemical entities and provide important ways to discover new drugs. Complementary and alternative medicine (CAM) can be used to maintain human health for a long time because of less side effects. In addition, an increasing number of patients with CVDs have resorted to the use of CAM, which shows a great application prospect and scope of exploration.

At present, in the exploration of natural drugs for the treatment of CVDs, including *Ligusticum chuanxiong* Hort. (Chuanxiong, CX) (Wang et al., 2018b), *Paeonia veitchii* Lynch. (Chishao, CS) (Ke et al., 2017), *Carthamus tinctorius* L. (Honghua, HH) (Jin et al., 2016), *Salvia miltiorrhiza* Bunge (Danshen, DS) (Lim et al., 2017), *Panax notoginseng* (Sanqi, SQ) (Fan et al., 2016), *Prunus persica* (L.) Batsch (Taoren, TR) (Li et al., 2020a), *Leonurus heterophyllus* Sweet (Yimucao, YMC) (Liu et al., 2019a), *Curcuma zedoaria* Roscoe (Ezhu, EZ) (Wu et al., 2019), etc., it has been found that they play a certain role in hemorheology (Wang et al., 2011), hemodynamics, platelet function (Chen et al., 2020), anticoagulation, antithrombotic (Zhou et al., 2016) and microcirculation functions, regulation of blood lipids (Xu et al., 2019), dilation of blood vessels (Yang et al., 2020a), regulation of energy metabolism (Ke et al., 2017), and other aspects. The above problems are often accompanied by the course of CVDs. These drugs focus on the whole in the prevention and treatment of complex diseases such as hypertension, showing the advantages of “multi-link and multi-target.” As one of the most frequently used drugs, CX can play its role in CVDs by expanding blood vessels and reducing the release of inflammatory mediators (He et al., 2018).

Evidence in the published literature suggests that CX is a herb with great potential to reduce the risk of CVDs and is already widely used clinically. Because CX has received good feedback in the treatment of CVDs, but there is no systematic summary of it, it is difficult to provide concise and clear reference significance for subsequent research and development. The purpose of this review is to summarize the current commonly used CVD drugs, and it has been found that CX has a huge competitive advantage in this aspect. Under the premise that the molecular mechanism of CX is still unclear, the material basis, pharmacological action, and clinical data of the cardiovascular and cerebrovascular aspects of CX are used as a support to provide a state-of-the-art overview of the chemistry and pharmacology about this valuable plant species and explore new perspectives and outline future challenges.

## 2 Characteristics of *Ligusticum chuanxiong* Hort.

*Ligusticum chuanxiong* Hort. (Chuanxiong), also known as *Ligusticum wallichii* Franchat, belongs to Angiospermae, family Umbelliferae, Ligusticum. It is a perennial herb with well-developed rhizomes and irregular nodules. The stem is upright, with vertical and longitudinal lines, many branches in the upper part, and the lower stem nodes expand into a disk shape. The lower part of the stem is petiolate, and the base expands into a sheath. Leaves are oval triangular, 12–15 cm long, and 10–15 cm wide, with 3–4 pinnate lobes. Compound umbellate catkin terminal or lateral seen (Song, 2015). It is cultivated in Sichuan, Guizhou, Yunnan, Guangxi, and other places in China, and its cultivation history can be traced back to 1,500 years ago (Yin, 2013). In addition, according to different producing areas, it can be divided into “Chuanxiong (CX)” produced in Sichuan, “Yunxiong (YX)” produced in Yunnan and Guizhou, “Dongxiong (DX)” produced in the East, and “Fuxiong (FX)” produced in the northeast (Liu, 2020).

As a natural drug, CX was introduced into Europe through Arabia in the 10th century. At present, it is used in more than 20 countries and regions and has become one of the important drug resources in the world, with Japan and South Korea as the main export places (Ma et al., 2001; Li and Yu, 2020). It is known as “Senkyu” (Shizu et al., 2012) and “Tousenkyu” (Yi et al., 2006) in Japan, “chungung” (Rhyu et al., 2004) in South Korea, “*Ligusticum chuanxiong* Hort.” (Lai, 2014) in Thailand, and “Szechuan lovage root” (Li et al., 2012) in Britain.

CX has always been used as medicine for its dry roots, but in recent years, it has been found that the stems, leaves, and fibrous roots of CX also have medicinal value. Compared with the rhizomes, only the content is different (Jiang et al., 2008). In the Bencao Huiyan, the aboveground part of CX is called “Mi Wu,” accounting for 75% of the fresh weight of the whole plant (Jiang et al., 2008; Tang et al., 2021). At the same time, CX is also one of the drugs that can be used for health food announced by the Ministry of Health of China in 2017 (Yu, 2017). In addition, CX is widely used in health-care products, food, cosmetics, and other fields. Its tender stems and leaves can be eaten as vegetables, and its rhizomes can also be stewed with beef and mutton for use (Yang et al., 2013). It has the characteristics of strong safety and wide application fields (Wen et al., 2019).

**FIGURE 1:**
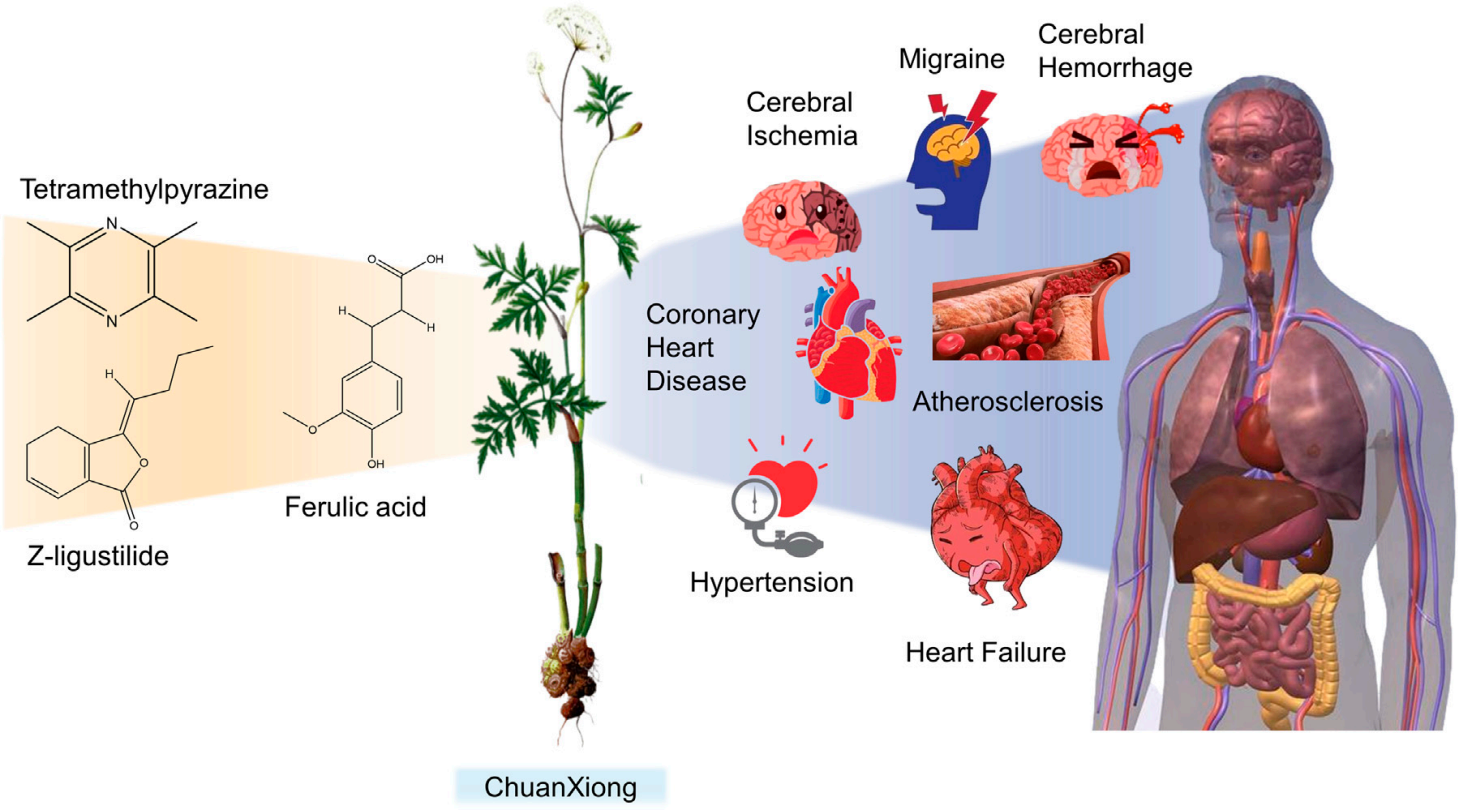
Basic information of *Ligusticum chuanxiong* Hort. (CX contains tetramethylpyrazine, Z-ligustilide, ferulic acid, and other components, which are widely used in CVDs, including hypertension, CHD, AS, heart failure, cerebral ischemia, and cerebral hemorrhage).

CX has been used by Chinese medicinal physicians for more than 2,000 years (Shan and Hao, 2011); its pharmacological effect was first mentioned in the Shen Nong Ben Cao Jing (a compilation of information regarding Chinese herbs dating back to 2800 BC) (Ran et al., 2011). It has therapeutic effects on the cardiovascular and cerebrovascular system, liver and kidney system, nervous system, respiratory system, urinary system, and many other systems (Zhang et al., 2020b). CX is widely used in CVDs, which provides a certain theoretical basis for solving the problem of high global mortality and disability (Figure 1). It is an effective drug for the treatment of CVDs. Moreover, CX is widely cultivated and inexpensive. Strengthening its application and development is conducive to reducing the economic burden of patients with CVDs.

## 3 Pharmacodynamic Substance Basis of *Ligusticum chuanxiong* Hort.

Up to now, more than 263 substances have been isolated from different parts (rhizomes, fibrous roots, and aboveground parts) of CX, such as volatile oils, organic acids, phenolic acids, phthalides, alkaloids, polysaccharides, ceramides, cerebrosides (Ran et al., 2011; Hung and Wu, 2016), and terpenoids (Chen et al., 2018). More than 80 compounds of which belong to various different structural types that have been identified (Li et al., 2012), including tetramethylpyrazine (TMP), ligustilide, senkyunolide A, ferulic acid, and other substances. At present, researches on active components of CX mainly focus on phthalides, alkaloids, and phenolic acids, while researches on other components are relatively few (Li, 2017). TMP and ferulic acid are the more functional and structural representatives of CX (Guo et al., 2016), among which TMP is the most active compound (Zhi-Gang et al., 2015). Based on the study of compounds isolated from CX, 42 compounds that have been proved to have CVD's protective effects are summarized in this article (Table 1).

**TABLE 1:** The pharmacodynamic material basis of *Ligusticum chuanxiong* Hort. in the treatment of CVDs.

Number	Component	Classification	Structural form	CAS	Molecular formula	Molecular weight	Reference
1	Z-Ligustilide	Volatile oil	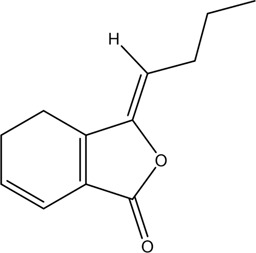	4431-01-0	C_12_H_14_O_2_	190.24 g/mol	Yi et al. (2006), Zhou et al. (2019b)
2	E-Ligustilide	Volatile oil	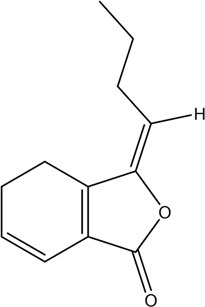	81944-08-3	C_12_H_14_O_2_	190.24 g/mol	Yi et al. (2006), Zhou et al. (2019b)
3	Senkyunolide A	Volatile oil	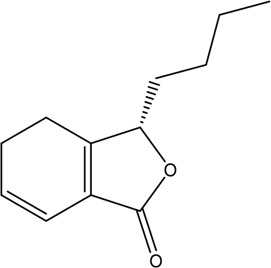	63038-10-8	C_12_H_16_O_2_	192.25 g/mol	Or et al. (2011), Zheng et al. (2018)
4	Senkyunolide H	Volatile oil	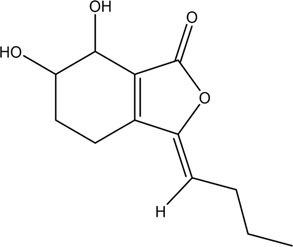	94596-27-7	C_12_H_16_O_4_	224.25 g/mol	Yi et al. (2006), Han et al. (2018)
5	Senkyunolide I	Volatile oil	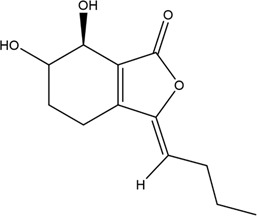	224.25 g/mol	C_12_H_16_O_4_	224.25 g/mol	Yi et al. (2006), Wang et al. (2012)
6	3-Butylidenephthalide	Volatile oil	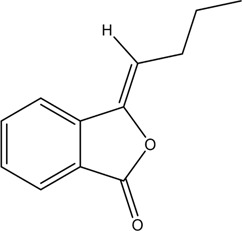	72917-31-8/551-08-6	C_12_H_12_O_2_	188.22 g/mol	Yi et al. (2006), Boo et al. (2009), Nam et al. (2013)
7	Butylphthalide	Volatile oil	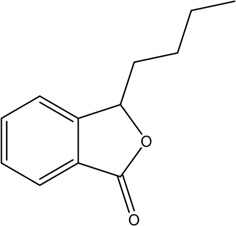	6066-49-5	C_12_H_14_O_2_	190.24 g/mol	Yi et al. (2006), He et al. (2021)
8	β-Elemene	Volatile oil	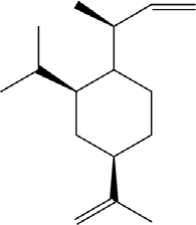	515-13-9	C_15_H_24_	204.35 g/mol	Liu et al. (2017b), Zhang et al. (2019)
9	Squalene	Volatile oil	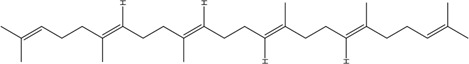	111-02-4	C_30_H_50_	410.7 g/mol	Zhang et al. (2019), 'Izzah Ibrahim et al. (2020)
10	α-Pinene	Volatile oil	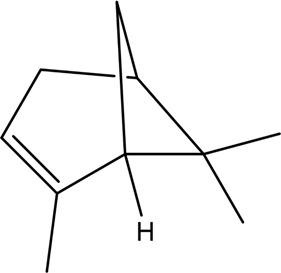	80-56-8	C_10_H_16_	136.23 g/mol	Zhang et al. (2019); Khoshnazar et al. (2020)
11	β-Myrcene	Volatile oil	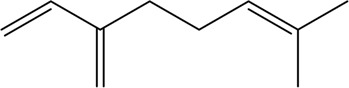	123-35-3	C_10_H_16_	136.23 g/mol	Burcu et al. (2016), Zhang et al. (2019)
12	Terpinen-4-ol	Volatile oil	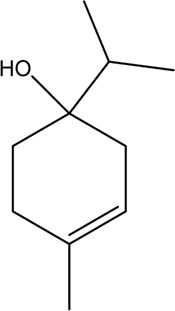	562-74-3	C_10_H_18_O	154.25 g/mol	Maia-Joca et al. (2014), Zhang et al. (2019)
13	Spathulenol	Volatile oil	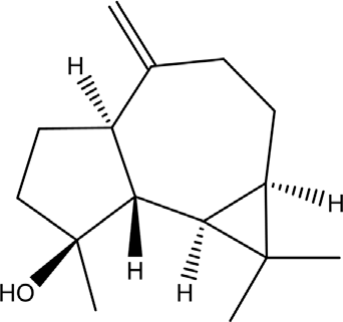	6750-60-3	C_15_H_24_O	220.35 g/mol	Suručić et al. (2017), Zhang et al. (2019)
14	Levistilide A	Volatile oil	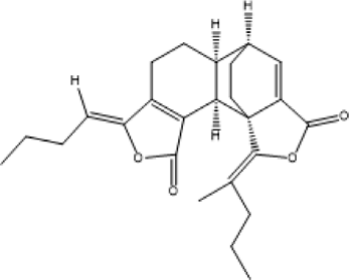	88182-33-6	C_24_H_28_O_4_	380.5 g/mol	Tong, (2021)
15	Ethyl ferulate	Volatile oil	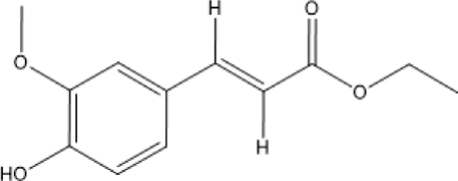	4046-02-0	C_12_H_14_O_4_	222.24 g/mol	Ya-Xian et al. (2021)
16	Terpinolene	Volatile oil	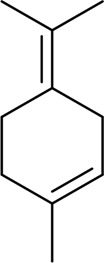	586-62-9	C_10_H_16_	136.23 g/mol	Labdelli et al. (2019)
17	Osthole	Volatile oil	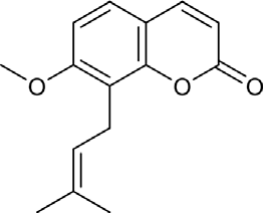	484-12-8	C_15_H_16_O_3_	224.28 g/mol	Shengzhong et al. (2019)
18	Neocnidilide	Volatile oil	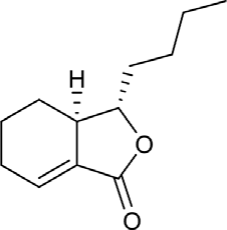	4567-33-3	C_12_H_18_O_2_	194.27 g/mol	Tang et al. (2021)
19	Z-6,7-Epoxyligustilide	Volatile oil	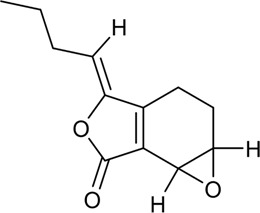	106533-40-8	C_12_H_14_O_3_	206.24 g/mol	Liu et al. (2004)
20	Marmesin	Volatile oil	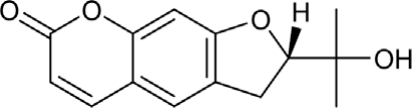	13848-08-6	C_14_H_28_O_2_	246.26 g/mol	Kim et al. (2015)
21	β-Sitosterol	Volatile oil	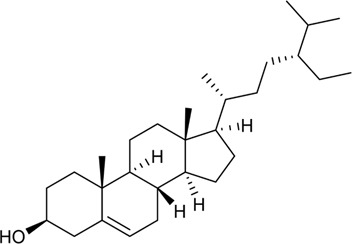	83-46-5	C_29_H_50_O	414.7 g/mol	Koc et al. (2020)
22	Wallichilide	Volatile oil	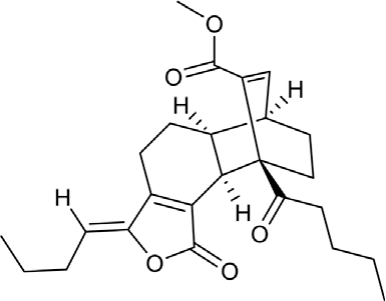	—	C_25_H_32_O_5_	412.5 g/mol	Kailin et al. (2021)
23	3-Carene	Volatile oil	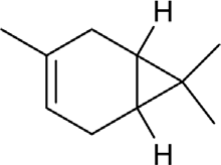	—	—	—	Sedigheh et al. (2013)
24	β-Eudesmol	Volatile oil	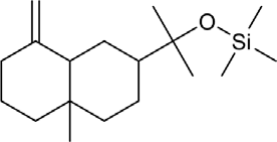	—	—	—	Acharya et al. (2021)
25	Tetramethylpyrazine	Alkaloid	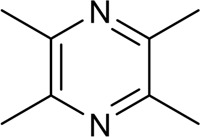	1124-11-4	C_8_H_12_N_2_	136.19 g/mol	Li and Chen (2004), Mi et al. (2020)
26	Adenosine	Alkaloid	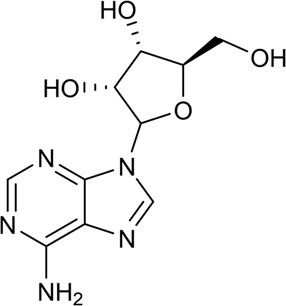	58-61-7	C_10_H_13_N_5_O_4_	267.24 g/mol	Guieu et al. (2020)
27	Inosine	Alkaloid	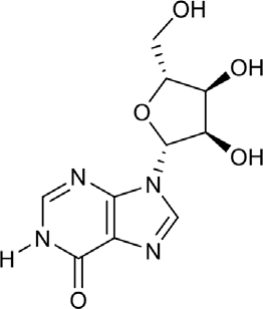	58-63-9	C_10_H_12_N_4_O_5_	268.63 g/mol	Lima et al. (2020)
28	Uridine	Alkaloid	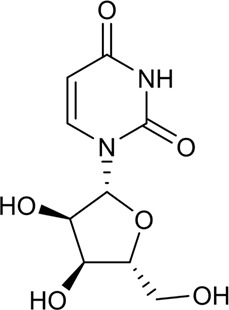	58-96-8	C_9_H_12_N_2_O_6_	244.2 g/mol	Krylova et al. (2021)
29	Choline	Alkaloid	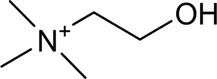	62-49-7	C_5_H_14_NO^+^	104.17 g/mol	Longzhu et al. (2017)
30	Ferulic acid	Phenolic acid	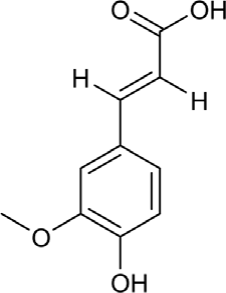	1135-24-6	C_10_H_10_O_4_	194.18 g/mol	Yi et al. (2006), Zhou et al. (2019b)
31	Neochlorogenic acid	Phenolic acid	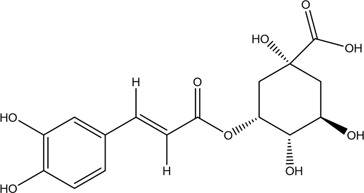	906-33-2	C_16_H_18_O_9_	354.31 g/mol	Liu et al. (2017a), Zhang et al. (2017)
32	Caffeic acid	Phenolic acid	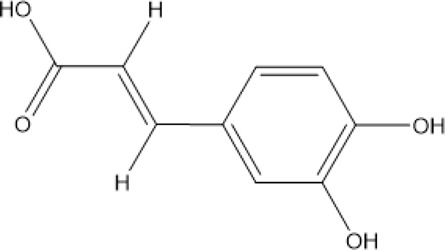	331-39-5/501-16-6	C_9_H_8_O_4_	180.16 g/mol	Gang (2018)
33	Folic acid	Phenolic acid	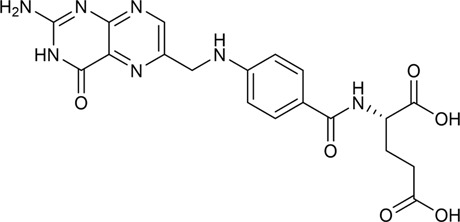	59-30-3	C_19_H_19_N_7_O_6_	441.4 g/mol	Ning et al. (2020)
34	Vanillic acid	Phenolic acid	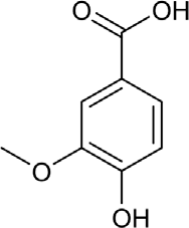	121-34-6	C_8_H_8_O_4_	168.15 g/mol	Xiuya et al. (2020)
35	Palmitic acid	Phenolic acid	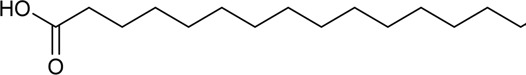	57-10-3	C_16_H_32_O_2_	256.42 g/mol	Haibo et al. (2021)
36	Vanillin	Phenolic acid	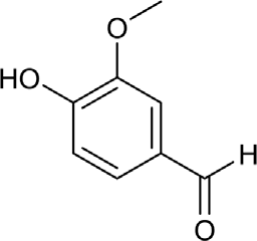	121-33-5	C_8_H_8_O_3_	152.15 g/mol	Sirangelo et al. (2020)
37	Tetradecanoic acid	Phenolic acid	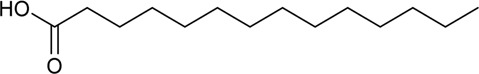	544-63-8	C_14_H_28_O_2_	228.37 g/mol	Oliviero et al. (2020)
38	Gallic acid	Phenolic acid	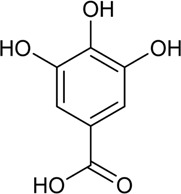	149-91-7	C_7_H_6_O_5_	170.12 g/mol	Jin et al. (2018)
39	Protocatechuic acid	Phenolic acid	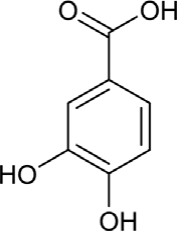	99-50-3	C_7_H_6_O_4_	154.12 g/mol	Liyan et al. (2021)
40	Chlorogenic	Phenolic acid	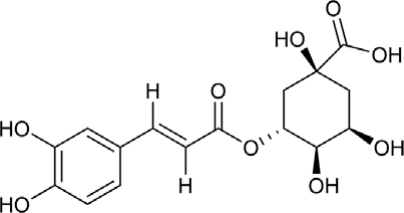	327-97-9	C_16_H_18_O_9_	354.31 g/mol	Bhandarkar et al. (2018)
41	Chrysophanic acid	Phenolic acid	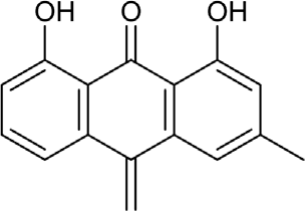	481-74-3	C_15_H_10_O_4_	254.24 g/mol	Lian et al. (2017)
42	Daucosterol	Others	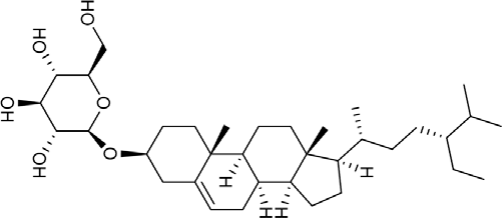	474-58-8	C_35_H_60_O_6_	576.8 g/mol	Wang et al. (2018)

### 3.1 Volatile Oil

CX contains 1% volatile oil, of which phthalides are the most important compounds, widely existing in *Angelica sinensis* (Oliv.) Diels (Danggui, DG), CX, and other umbelliferous plants, with low polarity and unstable heating characteristics (Fang et al., 2020). The phthalides in CX include more than 30 kinds of phthalides such as Z-ligustilide, E-ligustilide, senkyunolide A, and butylphthalide (Li et al., 2012). Modern pharmacological studies have found that phthalides have a variety of pharmacological activities, and the potential mechanism of cardio-cerebrovascular protection is related to the antithrombotic and antiplatelet effects, neuroprotective effects, improving blood fluidity, and inhibition of the abnormal proliferation of vascular smooth muscle cells (VSMCs) (León et al., 2017). Ligustilide is the most important phthalide in CX, which can play a neuroprotective role in cerebral ischemic diseases through its antioxidant and anti-apoptosis (Long et al., 2020). Butylphthalide was listed as one of the drugs that can be used in the treatment of ischemic stroke in 2005 by the food and Drug Administration of China (Zou et al., 2018).

### 3.2 Alkaloids

Up to now, 20 kinds of alkaloids have been found from CX, among which TMP is the most studied alkaloid, in addition to adenine, Senkyunolide A and other components (Pu et al., 2020a). TMP widely exists in Ligusticum species (Donkor et al., 2016). It was first extracted from CX in 1937 (Cai et al., 2014), and also used as one of the quality evaluation materials of CX (Pu et al., 2020a). It has been used effectively since the 1970s to treat ischemic heart disease, cerebrovascular and thrombotic vascular diseases (Hintz and Ren, 2003). Modern pharmacological studies have found that alkaloids have a variety of pharmacological activities. The potential mechanisms of cardio-cerebrovascular protection are related to antiplatelet aggregation, antithrombotic, antioxidant, anti-inflammatory, inhibition of abnormal proliferation of VSMCs, and neuroprotection (Pu et al., 2020b).

### 3.3 Phenolic Acids

The total phenolic acids of CX is the basis of the pharmacodynamic substance in CX, containing not less than 18 kinds of phenolic acids (Li et al., 2012), which are given priority to with ferulic and chlorogenic acids (Pu et al., 2018). Ferulic acid is an effective component in CX and DG (Luo et al., 2019a), and its content is high in CX. It is often used as the quality control index of CX (Zhang et al., 2018b). According to the Chinese Pharmacopoeia 2020, the content of ferulic acid in dried CX should not be less than 0.01%. Modern pharmacological studies have found that CX phenolic acid has a variety of pharmacological activities. The potential mechanism of cardio-cerebrovascular protection is related to neuroprotective effect and regulation of inflammatory metabolism (He et al., 2018; Bao et al., 2019).

### 3.4 Polysaccharides

Polysaccharides widely exist in higher animals, plants, algae, and fungi. As a kind of biological macromolecule, its biological activity is related to its complex structure (Zhang, 2011). Research on the chemical composition of CX has been carried out since the 1950s, while research on CX polysaccharide only started in 2008. CX polysaccharide is composed of glucose, galactose, arabinose, xylose, rhamnose, and mannose (Li et al., 2017a). Modern pharmacological studies have found that CX polysaccharide has a variety of pharmacological activities. The potential mechanism of cardio-cerebrovascular protection is related to antioxidant and anti-inflammatory (Zhao et al., 2017).

### 3.5 Other Categories

Glycosides, ceramides, cerebrosides, terpenoids, steroids, flavonoids, and other components were also found in CX (Li, 2017). Wang et al. (2018) found that daucosterol was one of the effective components in the treatment of cerebrovascular diseases when analyzing the active components of *Erigeron breviscapus* (Vant.) Hand.-Mazz.

## 4 Pharmacological Action of *Ligusticum chuanxiong* Hort.

**FIGURE 2:**
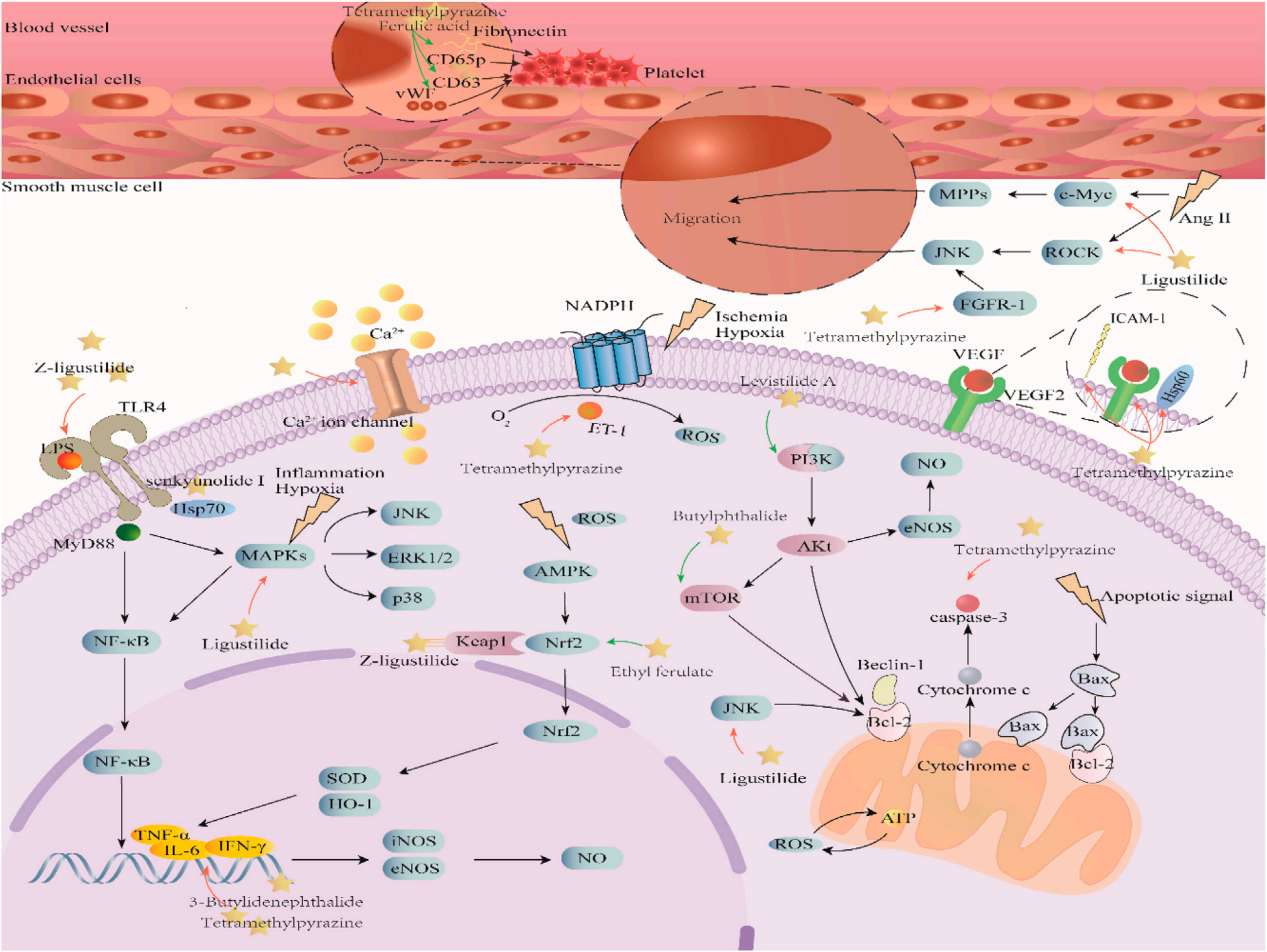
Mechanism and pathway of *Ligusticum chuanxiong* Hort. in the treatment of CVDs.

Better understanding of the CX effective components has revealed a complex regulatory mechanism, and these interactions are influenced by each other and form a wide, complex, and multi-directional regulatory network. These effects include anti-inflammatory, antioxidant, maintenance of endothelial cell stability, etc., which are usually associated with the course of CVDs. A systematic understanding of the mechanism of CX is conducive to guiding its clinical application. Here, we sorted out the complex network mechanism of CX in the CVDs, which is a powerful means to enrich our understanding of the “multi-link and multi-target” therapeutic mechanism of CX (Figure 2).

### 4.1 Inhibition of Inflammatory Mediators

Inflammation is a common pathophysiological sign of complex diseases, including AS and myocardial damage, that has recently emerged as an important contributor for CVDs development. In addition, it further exacerbates its harmful cardiovascular effects by interacting with the cardiovascular risk factors, thus creating a vicious cycle (Luca et al., 2020). The continuous increase of inflammatory mediators and the decrease of circulating anti-inflammatory cytokines may aggravate the remodeling of vascular extracellular matrix (ECM) and arteriosclerosis, thus expanding pulse pressure, promoting systolic blood pressure, and aggravating cardiovascular conditions (Hirofumi et al., 2017).

In CX, phthalides, such as senkyunolide A, senkyunolide I, and Z-ligustilide, ethyl ferulate, and other components can inhibit the expression of inflammatory factors and achieve anti-inflammatory effect. This effect may be realized through the Hsp70/TLR4/NF-κB, MAPK, and AMPK/Nrf2 pathways. The microglia are resident macrophages in the brain, which are activated during ischemia and produce various inflammatory mediators, including tumor necrosis factor-α (TNF-α), NO, interleukin-6 (IL-6), and interleukin-1 (IL-1). Among them, TNF-α induces post-stroke injury in a variety of ways (Or et al., 2011). Senkyunolide A and Z-ligustilide inhibit the expression of iNOS and TNF-α-mRNA in the microglia and protect neuro-2A cells from activated microglia-induced cytotoxicity (Or et al., 2011). The contents of NO, TNF-α, and interleukin-1 β (IL-1β) in the lipopolysaccharide (LPS)–induced rat brain microglia were decreased and the activation of the microglia was inhibited by butenylphthalide (Nam et al., 2013). Toll-like receptors (TLRs) are highly conserved members of the interleukin-1 receptor superfamilies, which can result in the activation of innate immune responses. Most TLRs are activated by the protein products produced by myeloid differentiation primary gene 88 (MyD88). Activation of the TLRs makes the nuclear factor kappa-B (NF-κB) activated, moves it to the nucleus, and initiates the transcription of immune-related genes, including interferon-γ (IFN- γ), IL-6, TNF-α, and so on (Mayra et al., 2015). The extracellular heat shock protein 70 (Hsp70) is an endogenous ligand of TLR4 and can inhibit TLR4 through negative regulation to play a protective role. Senkyunolide I inhibits oxygen glucose deprivation/reoxygenation–induced TLR4 elevation, and decreases MyD88 expression and NF-κB nuclear translocation. At the same time, the Hsp70 gene is silenced by siRNA before senkyunolide I treatment. It has been found that downregulation of Hsp70 enhanced the expression of TLR4 and weakened the inhibitory effect of senkyunolide I on NF-κB (Hu et al., 2016b). In addition, TLRs can also activate MAPK, and p38 mitogen-activated protein kinase (p38MAPK), c-Jun terminal kinases (JNKs), and ERK1/2 that have been identified as three major subgroups of MAPKs. p38 and JNK, as two major pathways of MAPK signal transduction, play an important role in inflammatory diseases (Zhou et al., 2019b). Hypoxia and inflammatory factors can activate the p38MAPK and JNK signaling pathways, but the downregulation of p38 MAPK and JNKs can reduce brain injury and neurologic deficit of focal cerebral ischemia and contribute to nerve protection (Gu et al., 2018). Ligustilide can reverse nitroprusside-induced activation of JNK and P38MAPK (Zhou et al., 2019b). In addition, adenosine monophosphate–activated protein kinase (AMPK) can reduce the inflammatory response, and ethyl ferulate can enhance the phosphorylation of AMPK, increase the expression of downstream Nrf2 gene, and promote its nuclear translocation. AMPK inhibitor could reverse the increase of Nrf2 expression caused by ethyl ferulate, and ethyl ferulate did not show the effect of increasing Nrf2 expression in Nrf2 knockout mice (Ya-Xian et al., 2021). The above data can well explain the anti-inflammatory effect of ethyl ferulate, which can be realized through activation of the AMPK/Nrf2 pathway.

### 4.2 Antioxidant

Oxidative stress (OS) greatly leads to endothelial dysfunction and increases the risk of cardiovascular and cerebrovascular events (Heitzer et al., 2001). Excessive reactive oxygen species (ROS) are its direct primers, including the forms of hydroxyl radical (OH), superoxide anion (O^2−^) and hydrogen peroxide (H_2_O_2_), which have become key mediators in the pathogenesis of vascular diseases such as stroke (Davis et al., 2019). Absolute and relative ischemia and hypoxia cause the decrease of oxygen supply and energy supply, which is the primary factor causing OS (Yang and Shanshan, 2015). Under the induction of cerebral ischemia, the energy consumption in the brain can promote the activity of OS. The highly activated nicotinamide adenine dinucleotide photosphate (NADPH) oxidase will produce excessive ROS to aggravate the damage (Ni et al., 2014). In addition, a large number of ROS can cause changes in mitochondrial membrane potential, enhancing its permeability and damaging mitochondria. The loss of ATP synthesis further exacerbates ROS production, creating a vicious cycle (Yang and Shanshan, 2015). Excessive ROS can cause fatal influence to the cells and organs, transforming the redox equilibrium state into a pro-oxidation state. ROS can block blood circulation, destroy epithelial cells and inhibit vasodilation by affecting blood vessels (Lin et al., 2019).

A variety of components in CX can exert antioxidant effects, which may be realized through the Nrf2 pathway and inhibition of NADPH enzyme. Superoxide dismutase (SOD) is an important enzyme for ROS removal in the body; the total antioxidant capacity (T-AOC) is the overall level of antioxidants in the body, which can reflect the antioxidant capacity of the body; malondialdehyde (MDA) is the final product of ROS lipid peroxidation, which can reflect the degree of lipid peroxidation. CX extract can reverse the decrease of SOD and T-AOC and the increase of MDA in the myocardial ischemia model, showing its antioxidant ability (Wang et al., 2017b). When OS occurs, the synthesis of redox sensitive transcription factor NF-E2–related factor 2 (Nrf2) is increased and Nrf2 is activated, increasing Nrf2 transfer from the cytoplasm to the nucleus. The upregulation of heme oxygenase-1 (HO-1) and SOD expression results in NF-κB failure and alleviates OS injury (Li et al., 2017b). Studies have shown that, as an antioxidant, TMP can exert its antioxidant effect by directly scavenging ROS or inhibiting the activity of the main source of ROS, namely NADPH oxidase (Jiang et al., 2011). Angiotensin II (AngII) can stimulate NADPH oxidase to produce ROS in VSMCs, which can be induced by endothelin-1 (ET-1). TMP can significantly inhibit the expression of ET-1 mRNA induced by AngII, reduce the activity of NADPH enzyme, and reduce the expression of ROS (Wong et al., 2007).

### 4.3 Protection of Endothelial Cells

Vascular events are caused by many factors of complex events. Endothelial cells play an important role in the regulation of vascular homeostasis and are closely related to the regulation of vascular tone, platelet activity, leukocyte adhesion, and thrombosis (Heitzer et al., 2001). Endothelial dysfunction is almost the common denominator of most vascular events (Ni et al., 2014), which is the earliest indicator of the development of cardiovascular disease. Vascular endothelium is a selective blood and tissue barrier that promotes angiogenesis and is necessary for tissues to adequately meet their metabolic and functional needs over the long term. Vascular endothelial cells (VECs) can promote angiogenesis and further aggravate inflammation-related injuries, causing plaque dilation, internal bleeding, and rupture, and promoting atherosclerotic lesions (Zhang et al., 2014; Wang et al., 2015). Therefore, inhibition of VECs growth can help treat AS.

TMP, levistilide A, and other components of CX play an important role in maintaining normal physiological functions of VECs, which may be achieved by inhibiting proliferation of endothelial cells, inhibiting the VEGF/VEGFR2 pathway, activating the PI3K-Akt-ENOS pathway, reducing ROS and icAM-1 and HSP60 expressions. Vascular endothelial growth factor (VEGF) is a key pro-angiogenic factor that promotes angiogenesis by activating VEGF2 receptors on the endothelial cells. Yuan et al. (2018) found that in the model of human umbilical vein endothelial cells (HUVECs) when induced by low concentrations of oxidized low-density lipoprotein (LDL), the number of vascular branch points increased and angiogenesis-related proteins VEGF and VEGF2 were upregulated. After TMP treatment, VEGF2 was downregulated, but VEGF was not affected. These data suggest that TMP can play a therapeutic role in reducing angiogenesis by inhibiting the VEGF/VEGFR2 pathway and inhibiting endothelial cell generation. NO is the key to maintaining endothelial function, and it can protect VECs by vasodilating in the physiological state. The activation of the PI3K-Akt pathway can affect the level of eNOS and thus increase the content of NO. Levistilide A can improve the continuity and integrity of damaged VECs and enhance their vitality. In addition to protecting VECs, the expression levels of PI3K, AKT, and eNOs proteins were increased (Tong, 2021). In addition, excessive ROS produced by VEC stress can also lead to endothelial dysfunction. O^2−^ is an ROS associated with the cytotoxicity of VECs, and H_2_O_2_ can produce O^2−^ by activating NADPH oxidase. Ni et al. (2014) found that TMP could improve Ach-induced relaxation of VECs and reverse O^2−^ induced by H_2_O_2_, which is consistent with the inhibition effect of NADPH oxidase. Under normal physiological functions, the expression levels of intracellular adhesion molecular-1 (ICAM-1) and heat shock protein 60 (HSP60) in VECs are low. However, when VECs are stimulated by inflammatory factors such as TNF-α, the expressions of ICAM-1 and HSP60 are upregulated, which induces the expression of cell adhesion molecules and produces autoantibodies and autoimmune responses, which are one of the pathogenesis of AS. Wu et al. (2012) detected the levels of ICAM-1 and HSP60 in HUVECs under the influence of TNF-α. It was found that the levels of ICAM-1 and HSP60 of HUVECs stimulated by TNF-α were significantly reduced before and after TMP administration, thus achieving protective effect against VECs disorders and reducing leukocyte adhesion.

### 4.4 Influence on Autophagy and Apoptosis

Apoptosis is a gene-controlled leukocyte death process—also known as programmed cell death under physiological conditions, is one of the mechanisms to maintain the stability of the internal environment, and is also an important mechanism of cerebral ischemia reperfusion and other diseases. Autophagy, also known as type ii programmed cell death, is often used as a protective mechanism. Under normal physiological conditions, it can remove damaged organelles, protein aggregates, and invading pathogens and maintain cell homeostasis. Under pathological conditions, the over-activation or inhibition of autophagy is often accompanied by a variety of diseases, including AS, myocardial ischemia–reperfusion injury, heart failure, and other cardiovascular diseases (Wang et al., 2017a; Demin et al., 2018). Autophagy and apoptosis are a double-edged sword and are balanced by Beclin-1 and Bcl-2. Beclin-1 can promote autophagy under the regulation of a variety of proteins, but this pro-autophagy binding can be blocked by anti-apoptotic Bcl-2 protein. Activation of PI3K/Akt phosphatidylinositol 3-kinase/serine/threonine kinase (PI3K/Akt) pathway can promote the interaction between Beclin-1 and Bcl-2 protein, thereby inhibiting autophagy (Wang et al., 2017a). Phosphorylation of Beclin-1 and Bcl-2 alone can lead to the separation of the two proteins, for example, phosphorylation of Bcl-2 by JNK promotes autophagy. In addition, JNK and P38 are major pathways of MAPK signaling and play important roles in stimulating apoptotic signals and inflammation. It has been found that Bcl-2 family plays an important role in apoptosis caused by mitochondrial dysfunction. Bax can promote apoptosis, and Bcl-2 can resist apoptosis (Jun et al., 2015). When the mitochondrial membrane potential changes, cytochrome c will be released to the cytoplasm, which stimulates Caspase-3. Bcl-2 located on the outer membrane of the mitochondria can prevent the release of pro-apoptotic proteins such as cytochrome c. After receiving the apoptosis signal, the inactive monomer protein Bax, which is free in the cytoplasm, moves to the outer membrane of the mitochondria and forms a channel, damaging the integrity of the mitochondrial membrane and antagonizing bcl-2 to prevent it from exerting the anti-apoptotic effect.

The volatile oil components in CX, including ligustilide, butylphthalide, senkyunolide A, H, and I, and TMP can play a therapeutic role in myocardial ischemia and cerebral ischemia–reperfusion injury by regulating autophagy and apoptosis. This role may be related to the PI3K/Akt/mTOR pathway, the MAPK pathway, and mitochondrial dependence. Cystine protease P32, also known as Caspase-3, is one of the most important pro-apoptotic factors of the Caspase family members and the common effector of multiple apoptotic pathways. Butylphthalide pretreatment can inhibit the expression of Caspase-3 and increase the level of p-Akt, an Akt phosphorylation product, in the brain of rats with cerebral ischemia–reperfusion injury (Yan, 2013). It shows that butylphthalide may achieve the effect of anti-apoptosis by activating the PI3K/Akt pathway. The mammalian target of rapamycin (mTOR) is a negative regulator of autophagy. Ligustilide can upregulate the expression of p-pI3k, p-Akt, and p-mTOR proteins in the brain of rats with cerebral ischemia–reperfusion and show the activation of the PI3K/Akt/mTOR pathway. It also upregulated Bcl-2 protein and downregulated the levels of pro-apoptotic protein Bax and Caspase-3 mRNA (Bo et al., 2021). The lactone components of CX (senkyunolide I: senkyunolide H: senkyunolide A: ligustilide = 1.00: 1.30: 6.90: 13.24) significantly increased the PI3K protein expression and Akt and mTOR protein phosphorylation levels, decreased Beclin-1 expression, and reduced the number of autophagic vesicles. The anti-autophagy effect of lactone components of CX can be terminated by PI3K inhibitor LY94002, which shows that it can play a role by activating PI3K/Akt/mTOR pathway (Gang, 2018). In addition, TMP downregulated the level of Caspase-3 and increased the ratio of Bcl-2/Bax. This effect can be reversed by LY294002, a specific inhibitor of the PI3K/Akt signaling pathway (Su et al., 2019). Ligustilide can increase the expression of Bcl-2 and reduce the levels of Caspase-3, Bax, and iNOS. The effect of ligustilide can be reversed by JNK and p38MAPK agonist anisomycin (Zhou et al., 2019b). This shows that the inhibitory effect of ligustilide on apoptosis may be achieved by inhibiting JNK and p38 MAPK. Xuesong et al. (2019) found that the ratio of cytochrome c and Cox IV in the mitochondria decreased and that in the cytoplasm increased in the model group. In addition, TUNEL and DAPI staining showed that the apoptosis in the model group reached 41%, accompanied by the upregulation of Caspase-3 and Bax/Bcl-2. TMP attenuated the change of mitochondrial membrane potential, inhibited the release of cytochrome c from the mitochondria to the cytoplasm, and decreased the expression of Caspase-3 and Bax/Bcl-2.

### 4.5 Inhibition of Smooth Muscle Cell Proliferation

VSMCs, endothelial cells, and ECM are the same components of the vascular wall. In line with endothelial cells, VSMCs can control the diameter of the lumen by coordinating the contraction and relaxation of the vessels, thus maintaining blood pressure (Wei and Wu, 2020). The proliferation, migration, and phenotypic transformation of VSMCs are one of the characteristics of vascular stenosis and the main pathological basis of vascular diseases. In addition, the phenotypic plasticity of VSMCs enables them to quickly adapt to environmental changes and plays an important role in CVDs (Park et al., 2019). NF-κB is a transcription factor commonly found in eukaryotes. When the body has an immune or stress response, NF-κB is activated and then transferred to the nucleus where it can stimulate or inhibit the transcription of some genes (Wei, 2020).

Ligustilide and TMP in CX can inhibit the proliferation of VSMCs and maintain the normal function of blood vessels. This effect may be related to JNK phosphorylation, and the c-Myc/MMP2 and ROCK/JNK pathway. Ligustilide can inhibit Ang Ⅱ–induced VSMC migration and downregulate migration-related proteins c-Myc, MMP2, ROCK1, ROCK2, p-JNK, and JNK (Luo et al., 2019b). The increased expression of fibroblast growth factor receptor-1 (FGFR-1) can activate the phosphorylation of JNK and affect the migration of VSMCs. TMP can reduce the proliferation of VSMCs induced by PM2.5, accompanied by the decrease of FGFR-1 and JNK phosphorylation levels. This effect is consistent with the results of JNK inhibitor treatment, suggesting that TMP can inhibit the proliferation of VSMCs by reducing the phosphorylation level of JNK (Qiang et al., 2017). c-Myc gene regulates cell proliferation and migration, and its high expression can enhance cell motility. The matrix metalloproteinases (MMPs) can degrade most of the components of the extracellular matrix, destroy the basement membrane formed during migration, and damage its migration inhibition, so as to make cells migrate smoothly. Ang II increased the expression of protein and mRNA of c-Myc and MMPs. Transwell cell migration experiment also found that cell migration was enhanced. The treatment with ligustilide and c-Myc inhibitor showed the downregulation of the expression of c-Myc and MMPs, accompanied by the decrease of cell migration. It can be preliminarily determined that ligustilide can inhibit the migration of VSMCs through the c-Myc/MMP2 signaling pathway. Rho-GTP has an important relationship with cell migration. Rhoo-associated kinase (ROCK) is the related kinase of Rho and is necessary to mediate the actin structure and contraction response of VSMCs. Ligustilide can inhibit the expression of ROCK1 and ROCK2 induced by Ang II, activate Rho/ROCK pathway, reduce the expression of JNK, and form signal interaction, but the role of them is not clear. After treatment with ROCK-specific inhibitors, it was found that the decrease of ROCK1 and ROCK2 was accompanied by the decrease of JNK expression, suggesting that ligustilide can inhibit the migration of VSMCs by regulating the ROCK/JNK signaling pathway (Luo et al., 2019b).

### 4.6 Adjust NO

Nitric oxide synthase (NOS) is the only rate-limiting enzyme for NO production. It can be divided into endothelial NOS (eNOS), neuronal NOS (nNOS), and inducible NOS (iNOS). eNOS is mainly expressed in endothelial cells. Under normal conditions, eNOS produces a small amount of NO, which can dilate blood vessels and has a neuroprotective effect. Under pathological conditions, nNOS and iNOS produce a large amount of NO, play a neurotoxic role, and cause cerebrovascular dysfunction.

TMP and other components in CX can regulate NO, which may be achieved by inhibiting NOS. And IL-1β, IL-2, IL-6, TNF-α, IFN-β, and other proinflammatory cytokines can induce high expression of iNOS. TMP can inhibit the expression of eNOS and iNOS induced by LPS to downregulate the level of NO in the rat brain. The expression of IL-6, TNF-α, and IL-1β was decreased (Sheng et al., 2020). It shows that CX can reduce the expression of NO by affecting proinflammatory cytokines and inhibiting NOS.

### 4.7 Influence on Calcium Ions

Intracellular Ca^2+^ homeostasis is necessary to maintain cell function. When cardiomyocytes are activated, changes in the cell membrane potential will open up L-type calcium current (ICA-L) and increase the amount of Ca^2+^ entering the cytoplasm from the outside of the cell. Large amounts of Ca^2+^ will bind to troponin, causing excitatory contraction of the myocardium and myocardial injury (Zhao et al., 2020). Therefore, calcium overload can be alleviated by inhibiting calcium channels and intracellular Ca^2+^ homeostasis can be maintained to achieve cardiac protection.

The regulating effect of TMP and other components in CX on calcium ions may be related to regulating calcium channels. TMP reduces Ca^2+^ increase in cells by selectively opening KATP or SKCa channels (Wong et al., 2003). Wu et al. (1989) found that at low doses, TMP predominantly prevents the mobilization of intracellular calcium, but at a higher dose, it inhibits the influx of extracellular calcium. Ren et al. (2012) found that TMP can dose-dependently reduce the ICA-L of rabbit cardiomyocytes, and this inhibition can be restored after elimination of the drug. At the same time, TMP can inhibit the transient and contraction of calcium in rabbit cardiomyocytes under physiological and pathological conditions.

### 4.8 Anti-Platelet Aggregation

Platelet activation and aggregation play a crucial role in AS. Platelet proteomics revealed that platelet gelsolin is a differential functional protein between patients with CHD and healthy people and is highly expressed in patients (Liu et al., 2013). In addition, platelet aggregation also plays an important role in thrombosis, which is related to AS, stroke, and various inflammatory reactions. Inhibition of platelet aggregation can effectively prevent and slow down the formation of thrombosis (Chen et al., 2017a).

TMP, ferulic acid, and other components in CX can inhibit platelet aggregation, which may be achieved by reducing platelet activation and the expression of adhesion molecules. TMP was used to treat patients with acute coronary syndrome (ACS) after percutaneous coronary intervention (PCI). It was found that the two platelet activation indexes of CD65p and CD63 decreased significantly before and after the treatment (Xu and Li, 2019). TMP can reduce the expression of two adhesion molecules the von Willebrant Factor (vWF) and fibronectin in endothelial cells, inhibit platelet activation and aggregation, promote blood circulation and reduce platelet adhesion (Wang et al., 2016).

Xu and Li (2019) used TMP to treat ACS patients after PCI using CD65p, CD63 (two platelet activation markers), platelet adhesion test (PADT), and gelsolin to evaluate the therapeutic effect of postoperative antiplatelet aggregation. It was found that the differences of CD65p, CD63, PADT, and gelsolin before and after operation in the TMP treatment group was greater than in the control group, which showed that TMP could significantly inhibit platelet activation. Wang et al. (2016) used TMP in the treatment of allergic asthma rats induced by ovalbumin and found that the adhesion of platelet to HUVECs was inhibited by TMP, and the expression of two adhesion molecules, vWF and fibronectin, expressed in endothelial cells, was reduced in the asthmatic rats treated with TMP. It is confirmed that TMP markedly inhibited platelets activation and aggregation, and promoted blood circulation, and platelet adhesion was significantly attenuated. Zhang et al. (2020a) demonstrated that TMP can partially inhibit the p38MAPK and NF-κB signaling pathways, thereby reducing platelet adhesion of mouse micro-VECs induced by oxygen–glucose deprivation/reoxygenation injury. Choi et al. (2018) through the study of ferulic acid in *in vivo* and *in vitro* experiments found that ferulic acid can inhibit platelet aggregation to play a role in weakening platelet activation by stimulation and can play an antithrombotic role in the acute thromboembolism model.

## 5 *Ligusticum chuanxiong* Hort. in the Treatment of Cardio-Cerebrovascular Diseases

Under the guidance of the current situation that CX is widely used in CVDs, the effects of CX on hypertension, CHD, AS, heart failure, cerebral hemorrhage, and cerebral ischemia are described. Supported by clinical data and combined with network pharmacology research, the signaling pathway and mechanism of CX in the treatment of CVDs are constantly explored to promote its more comprehensive application.

### 5.1 Cardiovascular Disease

#### 5.1.1 Hypertension

According to the American Heart Association, hypertension is one of the five major risk factors for disease burden in areas other than Oceania and the eastern, central, and western parts of sub-Saharan Africa (Virani et al., 2021). It is one of the most important risk factors for CVDs and is closely related to stroke, heart failure, and other CVDs. Currently, the prevalence rate of hypertension in China is 50% and the United States is 46% (Chen et al., 2019a).

Through the analysis of network pharmacology of CX and hypertension, it was found that CX could be based on the calcium signaling, VEGF signaling, PI3K-Akt signaling, cGMP-PKG signaling, TNF signaling, and estrogen, insulin resistance, and prostate cancer pathways. CX can be involved in the treatment of hypertension by blocking vasoconstrictor molecules, increasing the number of smooth muscle cells, reducing the number of intracellular Ca^2+^ and other mechanisms (Yan et al., 2020).

According to the statistics, Tian et al. (2021) analyzed the clinical data of TCM in the treatment of hypertension patients and found that among the hypertension diseases with different syndrome types, CX was used the most frequently, and according to different syndrome types, it was combined with *Gastrodia elata* Blume (Tianma, TM), *Dioscorea opposita* Thumb. (Shanyao, SY), DG, *Pinellia ternate* (Thunb.) Breit. (Banxia, BX), etc.

#### 5.1.2 Coronary Heart Disease

CHD, also known as coronary atherosclerotic heart disease, is a kind of heart disease caused by myocardial ischemia, hypoxia, or necrosis caused by coronary artery stenosis, spasm, or occlusion (Therapy, 2021). Angina pectoris refers to chest discomfort caused in the heart (Joseph and Colin, 2020), which is one of the common complications of patients with CHD. About 80% of patients with CHD will have angina pectoris (Wei, 2021).

Through the analysis of network pharmacology of CX in the treatment of CHD, seven compounds were selected from CX. It was found that they could be used in 31 targets such as vascular hemophiliac molecule, coagulation factor 2, transmembrane receptor protein 1, cardiac cell enhancement factor 2A, SOD 1, nitric oxide synthase 2, etc. Based on these targets, CX can play the role of anti-oxidation, anti-inflammation, anticoagulation, promoting angiogenesis, dilating blood vessels, regulating blood pressure, and other functions to realize the treatment of CHD (Zhao-et al., 2019).

Bi et al. (2020) investigated the clinical medication data of 1986 patients with CHD in 11 provinces, cities, and autonomous regions of China. Among them, drugs for property of activating blood and resolving stasis were used 2,173 times. In addition, in the application of single drug in the treatment of CHD, the frequency of use of CX reached 447 times, 56.58%, second only to Danshen. Ma (2018b) investigated the clinical efficacy of CX injection in patients with CHD and angina pectoris. The control group received angiotensin II receptor antagonists (ARBs), aspirin, nitrate drugs, and β-receptor blockers (BB), while the observation group was given ligustrazine injection on the basis of the control group. The clinical results showed that the frequency and duration of angina pectoris in the observation group were significantly lower than in the control group, and the total effective rate in the observation group (94.44%) was significantly higher than that of the control group (82.22%). As far as the clinical data of ligustrazine injection are concerned, they show that ligustrazine injection has a better disease control effect.

#### 5.1.3 Atherosclerosis

AS is the main pathological basis of cardiovascular disease. It refers to a progressive pathological process in which the structure or function of the endothelial cells of the large and medium arteries are damaged, the permeability of the intima is increased, and cholesterol and cholesterol lipid accumulate in or under the intima of the artery under the combined action of a variety of risk factors (Ma, 2016). The potential mechanisms include endothelial dysfunction, lipid deposition, OS injury, immune inflammatory response, and platelet migration and aggregation (Xin et al., 2020).

Through the analysis of network pharmacology of CX in the treatment of AS, 20 compounds were screened from CX and 37 targets were found in the treatment of AS. Through KEGG analysis of these targets, it was found that CX could exert anti-inflammatory, anti-OS, protect endothelial cells, inhibit the proliferation and migration of VSMCs, improve vasoconstriction, and inhibit platelet aggregation by affecting 14 pathways, including TNF, insulin resistance, vascular smooth muscle contraction, calcium ion, VEGF, TRP channel regulated by inflammatory mediators, and platelet activation, so as to achieve the therapeutic effect on AS (Tian et al., 2018).

Deng et al. (2017) analyzed the clinical efficacy of Danshen combined with CX in the treatment of elderly patients with AS. The control group was given routine treatment of AS, and the observation group was given Danshen: CX decoction (5:4). The levels of inflammatory factors and blood lipids in the venous blood of the patients were detected after 30 days. The levels of inflammatory factors including C-reactive protein (CRP), IL-1β, IL-6, TNF-α, monocyte chemotactic protein-1, and blood lipids total, including total cholesterol (TC), triglyceride, high-density lipoprotein cholesterol, and LDL cholesterol in the venous blood of the patients after 30 days were measured. It was found that there was no significant difference in the blood lipid level between the control and observation groups, whereas the inflammatory factor level of the observation group was significantly lower than that of the control group. The data showed that Danshen combined with CX had an obvious anti-infection effect in the treatment of elderly patients with AS and a better disease control effect on patients with AS. Zuo et al. (2020) studied the clinical efficacy of Xingnao Zhitan capsule (including CX) in the treatment of the carotid artery in patients with acute cerebral infarction. During the 3-month treatment, the control group was given atorvastatin calcium tablets and the observation group was given atorvastatin calcium tablets combined with Xingnao Zhitan capsule. After research, it was found that TC and LDL in the observation group were significantly lower than those in the control group, and FIB, D-D, hs CRP, IMT, Crouse scores were significantly improved, and the total effective rate in the observation group (82.1%) was significantly higher than that in the control group (60.5%). According to the analysis of the clinical data, the capsule can control AS by regulating blood lipid and reducing inflammation and plate volume.

#### 5.1.4 Heart Failure

Chronic heart failure (CHF) is a clinical syndrome with abnormal cardiac structure and function caused by a variety of risk factors (Jia et al., 2020b). It has the characteristics of high incidence rate, high mortality rate, and high medical expenditure (Li et al., 2020b). Only in the United States, heart failure accounted for 10% of the total medical expenses in medical spending, and patients with heart failure in 2030 are expected to increase to eight million.

Through the analysis of network pharmacology of CX in the treatment of heart failure, it was found that CX could realize the therapeutic effect of heart failure through 12 targets, one of which was related to chest and flank prickling pain. Through KEGG enrichment analysis of these targets, it has been found that CX can activate the cancer signaling pathway, apoptosis, HIF-1, and the other signaling pathways to achieve the functions of inhibiting inflammation, regulating apoptosis, and improving vasoconstriction to treat heart failure (Jia et al., 2020a).

Zhang (2019) analyzed the clinical efficacy of Danshen–ligustrazin for injection in the treatment of heart failure. The control group was given conventional treatment methods such as oxygen inhalation, cardiotonic, low salt diet, vasodilation, and bed rest. The observation group was given Danshen–ligustrazin for injection on the basis of the control group. After 14 days of treatment, the relevant levels of the patients were detected. It was found that the left ventricular ejection fraction (LVEF) of the observation group was significantly higher than that of the control group, and the brain natriuretic peptide (BNP) level was significantly lower. The total effective rate in the observation group (90%) was significantly higher than that in the control group (78%). Through clinical data analysis, Danshen–ligustrazin for injection can effectively improve cardiac function and hemodynamics and has a good control effect on heart failure. Sui et al. (2020) analyzed the clinical efficacy of Danshen–ligustrazin for injection in the treatment of blood stasis syndrome of CHF. The control group received conventional treatment of heart failure by limiting salt and water, using digitalis preparation, BB, angiotensin converting enzyme inhibitors (ACEI)/ARB, and aldosterone receptor antagonist and diuretic, and the observation group was given Danshen–ligustrazin for injection on the basis of the control group. It was found that compared with the control group, the levels of NT-proBNP and myocardial energy consumption were significantly decreased and LVEF was significantly increased in the observation group. In addition, the total effective rate of the observation group (80%) was significantly higher than that of the control group (57.17%). Through the analysis of clinical data, Danshen–ligustrazin for injection can relieve the clinical symptoms of CHF patients with blood stasis and improve cardiac function, so as to play a good therapeutic effect on heart failure.

### 5.2 Cerebrovascular Disease

In 2017, the Cerebrovascular Division of the Neurology Society of the Chinese Medical Association released the “Classification of Cerebrovascular Diseases in China 2015,” which explained cerebrovascular diseases and believed that cerebrovascular diseases are transient or permanent neurological dysfunction caused by various risk factors. Stroke is the second leading cause of death worldwide and increases the global medical burden (Shakiba et al., 2021), especially acute cerebrovascular disease, which is a focal vasogenic neurological deficit syndrome that can lead to death or permanent neurological defects (Fan, 2018). Stroke is characterized by high morbidity, high disability, high mortality, and high recurrence rate (Jin et al., 2021), including ischemic cerebrovascular disease and hemorrhagic cerebrovascular disease (Chen et al., 2017b). Ischemic strokes are more common, accounting for 87% of strokes in the United States. In Asian countries, cerebral hemorrhage accounts for 24–55% of all strokes, while in China, the rate is 24%, significantly higher than in developed countries (Li, 2019). Ischemic stroke refers to the blood clots in the blood that stop enough blood supply to the brain, and hemorrhagic stroke refers to the primary non-traumatic hemorrhage that cause bleeding within the brain parenchyma of the microvascular burst (Zheng and Deng, 2020). Hemorrhagic stroke is gender related, with women having a higher rate of stroke-related deaths than do men, averaging six out of 10 women (Derek and Hiranmoy, 2020).

#### 5.2.1 Cerebral Ischemia

Song et al. (2015) analyzed the material basis and molecular mechanism of CX in the treatment of cerebral ischemia through molecular docking technology. Through molecular docking of 45 components selected from CX with cerebral ischemia target proteins of four existing drugs on the market, it was found that 12 components of CX scored higher than the existing drugs on the market, and ferulic acid scored the highest. Other studies have proven that CX can inhibit the expression of Uba3a, thereby inhibiting the NF-κB signaling pathway and relieving neuron cell damage (Yu et al., 2017). Z-ligustilide nasal administration can prevent cerebral ischemia through the Nrf2 and HSP70 signaling pathways (Li et al., 2017b).

Cheng and Luo (2020) analyzed the clinical effect of TMP on elderly patients with early cerebral ischemia after intracranial aneurysm clipping. The control group was given edaravone injection, and the observation group was given TMP injection. After 14 days of treatment, it was found that the Barthel Index (BI) and SOD level of the observation group were higher than those of the control group, and the NIHSS score and plasma MDA level of the observation group were lower than those of the control group. The clinical data show that TMP can improve OS and reduce rebleeding in elderly patients with early cerebral ischemia after intracranial aneurysm clipping, which has important clinical value.

#### 5.2.2 Cerebral Hemorrhage

In the treatment of intracerebral hemorrhage, studies have shown that Danshen–ligustrazin for injection can reduce cell apoptosis by reducing the release of IL-6 and TNF-α, increasing the expression of NOS, decreasing the expression of Caspase-3, and increasing the expression of Bcl-2, thus playing a role in the treatment of intracerebral hemorrhage (Zhang et al., 2018a).

Sun et al. (2008) analyzed the clinical efficacy of ligusticum (sodium ferulic acid) for intracerebral hemorrhage. The control group was given routine treatments such as dehydration, brain cell activator, regulation of blood pressure and blood glucose, and management of stress complications, and the observation group was given intravenous infusion of ligusticum on the basis of the control group. Zhao (2020) analyzed the clinical efficacy of Taoren–Chuanxiong decoction on cerebral hemorrhage. The control group was treated with Piracetam tablet, and the observation group was treated with Taoren–Chuanxiong decoction on the basis of the control group. It was found that the whole blood high-shear viscosity, whole blood low-shear viscosity, plasma viscosity, and HCT of the observation group were significantly lower than those of the control group, and the NHISS, FMA, and BI scores of the patients were better than those of the control group, and the total effective rate in the observation group (88.89%) was significantly higher than that in the control group (71.11%). This shows that Taoren–Chuanxiong decoction has an obvious improvement effect on the nerve function defect and hemodynamics of the patients and can effectively control the condition of the patients with cerebral hemorrhage.

## 6 Combined Application of *Ligusticum chuanxiong* Hort. Against Cardio-Cerebrovascular Disease

### 6.1 Study on the mechanism of compound containing *Ligusticum chuanxiong* Hort.

So far, CX has been widely used in cardiovascular and cerebrovascular diseases and together with a variety of drugs, which provide a certain idea and feasibility for the prevention and treatment of CVDs and also a new direction for drug research and development. Modern pharmacological studies have found that it can reduce arterial resistance, increase cerebral blood flow, improve microcirculation, reduce capillary permeability, and has a protective effect on brain injury, so it plays an important role in the prevention and treatment of CVDs (Zeng et al., 2013). In previous studies, CX and other drug combinations were collected and identified for CVDs therapy, in order to provide theoretical support for the subsequent studies on CX resistance to CVDs (Table 2).

**TABLE 2:** Application of classical prescription containing *Ligusticum chuanxiong* Hort. in CVDs.

Classics	Prescription	Therapeutic effect	Indication	Reference
Golden Mirror of Medicine	Taohong Siwu decoction	1. Reduce the area of cerebral infarction and reduce neurological damage; 2. Promote blood circulation; 3. Promote angiogenesis and reduce apoptosis; 4. Anticoagulation and lipid lowering; 5. Inhibit apoptosis; 6. Reduce myocardial fibrosis	1. Cerebral ischemia–reperfusion injury; 2. Acute blood stasis; 3. Myocardial infarction; 4. CHD; 5. Middle cerebral artery occlusion reperfusion; 6. Myocardial infarction	Li et al. (2015b), Tan et al. (2021)
Correction on Errors in Medical Classics	Buyang Huanwu decoction	1. Inhibit the proliferation of VSMCs; 2. Improve hemorheological disorders and energy metabolism; 3. Neuroprotective effect; 4. Reduce and inhibit excitatory amino acids; 5. Improve synaptic plasticity and promote nerve repair; 6. Protect blood–brain barrier	1. CHD; 2. Ischemic stroke	Chen et al. (2011), Pan et al. (2017), Chen et al. (2019c)
Correction on Errors in Medical Classics	Decoction for activating blood circulation	Dilate blood vessels, improve microenvironment, and inhibit inflammatory reaction	1. Cerebral infarction; 2. Hypertension	Yang (2020b)
Danxi's Mastery of Medicine	Yueju pill	1. Improve myocardial tissue antioxidant; 2. Reduce inflammatory factors	1. Myocardial ischemia; 2. Angina pectoris; 3. Hypertension; 4. Hyperlipidemia; 5, CHD	Gui et al. (2016), Hu et al. (2017)
Essential Recipes for Emergent Use Worth A Thousand Gold	Xiao Xu Ming decoction	1. Inhibit autophagy-related protein and promote the recovery of neural function; 2. Reduce the damage of blood–brain barrier and cerebral ischemia; 3. Improve cerebral artery blood supply and hemodynamics, and promote the recovery of neural function; 4. Improve hemorheology, reduce blood viscosity, and improve the increase of cell aggregation; 5. Dilate blood vessels, inhibit vascular motor left, or sympathetic nervous system	1. Cerebral ischemia–reperfusion; 2. Acute cerebral infarction; 3. Hyperlipidemia; 4. Hypertension	Lan et al. (2013), Cheng et al. (2019), Wu and Wang (2020)
Clear Synopsis on Recipes	Dachuanxiong pill	1. Regulate hemorheology and hemodynamics; 2. Upregulate the level of VEGF	Cerebral ischemia	Zhang et al. (2006)
Correction on Errors in Medical Classics	Xuefu Zhuyu decoction	1. Inhibit inflammation and inhibit apoptosis; 2. Improve blood lipid and inhibit AS; 3. Improve cerebral infarction, improve neurological deficit; 4. Reverse myocardial fibrosis; 5. Reduce blood lipid and whole blood viscosity, improve heart energy supply, and regulate amino acid metabolism; 6. Improve vascular endothelial function, promote angiogenesis, and improve hemorheology	1. Thromboembolic stroke; 2. Hyperlipidemia; 3. Acute ischemic stroke; 4. Hypertension; 5. CHD; 6. AS	Lee et al. (2011), Shen et al. (2015)
Clear Synopsis on Recipes	Miraculous power of *Ledebouriella*	Promote and improve the level of lipid metabolism, and improve and regulate lipid metabolism and disorder	Hyperlipidemia	Xing (2002)
Prescriptions of the Bureau of Taiping People's Welfare Pharmacy	Decoction of 10 powerful tonics	Reduce the load of heart, improve the congestion of organs	1. Heart failure after acute myocardial infarction; 2. CHF	Pan and Liu, (2017), Yang (2020a)
General principles of Medicine	Chaihu Shugan powder	1. Reduce neurogenic inflammation; 2. Improve heart function and inflammatory reaction, and reduce adverse reactions	1. Stable angina pectoris of CHD; 2. Hypertension; 3. Acute myocardial infarction	Hu et al. (2016a), Yang et al. (2020b)
Correction on Errors in Medical Classics	Infradiaphragmatic stasis-expelling decoction	1. Improve hemorheology, reduce vascular resistance, inhibit platelet aggregation and release, increase plasmin activity, promote fibrinolysis, improve microcirculation; 2. Improve platelet aggregation, reduce inflammatory reaction, and myocardial injury	1. Angina pectoris of CHD; 2. Myocardial infarction	Liu et al. (2012), Liu et al. (2018)
Synopsis of Golden Chamber	Danggui Shaoyao powder	1. Improve the level of lipid peroxidation and hemorheology; 2. Improve the level of blood lipids; 3. Reduce the concentration of vasoconstrictors; 4. Increase the blood supply to the brain	1. Hyperlipidemia; 2. AS; 3. Hypertension; 4. Acute myocardial infarction	Ren et al. (2017)
SuWenBingJiQiYiBaoMingJi	Daqinjiao decoction	1. Improve blood pressure level and blood pressure rhythm; 2. Improve hemorheology and nerve function; 3. Improve the degree of nerve function defect	1. Hypertension; 2. Acute cerebral infarction; 3. Acute ischemic stroke	Song et al. (2018), Gao et al. (2019)

### 6.2 Modern Application of Preparations Containing *Ligusticum chuanxiong* Hort.

Compared with chemical medicine, natural medicine has the advantages of less side effects, multiple links, and multiple targets. Its preparation into capsules, granules, pills, tablets, oral liquid, injection, and other dosage forms is conducive to improve its bioavailability, enhances the convenience in its production, transportation, taking, carrying, and storage, and makes it better in playing its pharmacodynamic role. A total of 1640 modern preparations have been collected in the Chinese Pharmacopoeia, of which 9.21% are CX (Li, 2017). This article summarizes the current modern preparations containing CX, and their applications in CVDs are counted, in order to provide some reference for follow-up research of other scholars (Table 3).

**TABLE 3:** Application of *Ligusticum chuanxiong* Hort. preparation in CVDs.

Category	Name	Indication	Reference
Capsule	Compound Chuanxiong capsule	AS	Kang et al. (2015)
	Xinnaokang capsule	Angina pectoris	Li et al. (2019)
	Naoxintong capsule	Heart failure, myocardial infarction, cerebral ischemia–reperfusion, cerebral infarction	Wang et al. (2017c)
	Niuhuang Jiangya capsule	Hypertension	Ma (2018a)
	Zhengxin Tai capsule	Angina pectoris	Zhang and Qi (2015)
	Xueshuan Xinmaining capsule	Acute cerebral infarction	Ma et al. (2019)
	Xuemaitong capsule	Cerebral hemorrhage, acute myocardial infarction, AS, CHD, and angina pectoris	Wang et al. (2006), Ye et al. (2013)
	Shuxin Tongmai capsule	CHD	Zhang and Gao (2019)
	Guanxin Kang capsule	Heart failure, acute myocardial ischemia, CHD, and angina pectoris	Pan et al. (2007)
Granule	Mailuotong granules	Cerebral infarction	Wang et al. (2020)
	Zhengxintai granules	Angina pectoris	Li et al. (2005)
	Yixin Tongmai granules	Angina pectoris	Wang and Shi (2015)
	Guanxin Kang granules	Angina pectoris	Liu (2011)
Pill	Guanxin pill	Angina pectoris	Feng (2006)
	Angong Jiangya pill	Hypertension	Li and Wang (2019)
	Suxiao Jiuxin pill	AS, acute myocardial ischemia, CHD, myocardial infarction	Li et al. (2011)
	Niuhuang Jiangya pills	Hypertension	Yin (2018)
Tablet	ShuXinNing tablets	Hypertension, high cholesterol, CHD, angina pectoris	Wang et al. (2018a)
	Zhengxintai tablets	Angina pectoris	Xu (2007)
	Xiaoshuan Tongluo tablets	Focal cerebral ischemia	Li et al. (2016)
	Xueshuan Xinmaining tablets	CHD angina, CHD	Tan et al. (2018), Shan and Leng (2019)
Oral liquid	Guanxin'an oral liquid	Myocardial ischemia	Wang et al. (2005)
	Ruanmailing oral liquid	AS	Wu et al. (2013)
	Tongtian oral liquid	Acute cerebral infarction	Chen et al. (2019b)
Injection	Guanxining injection	Myocardial ischemia–reperfusion, CHD, angina pectoris, ischemic stroke	Xiao et al. (2021)
	Danshen ligustrazin for injection	Myocardial ischemia, cerebral ischemia–reperfusion injury	Huang et al. (2016)

## 7 The Security Toxicity of *Ligusticum chuanxiong* Hort.

**FIGURE 3:**
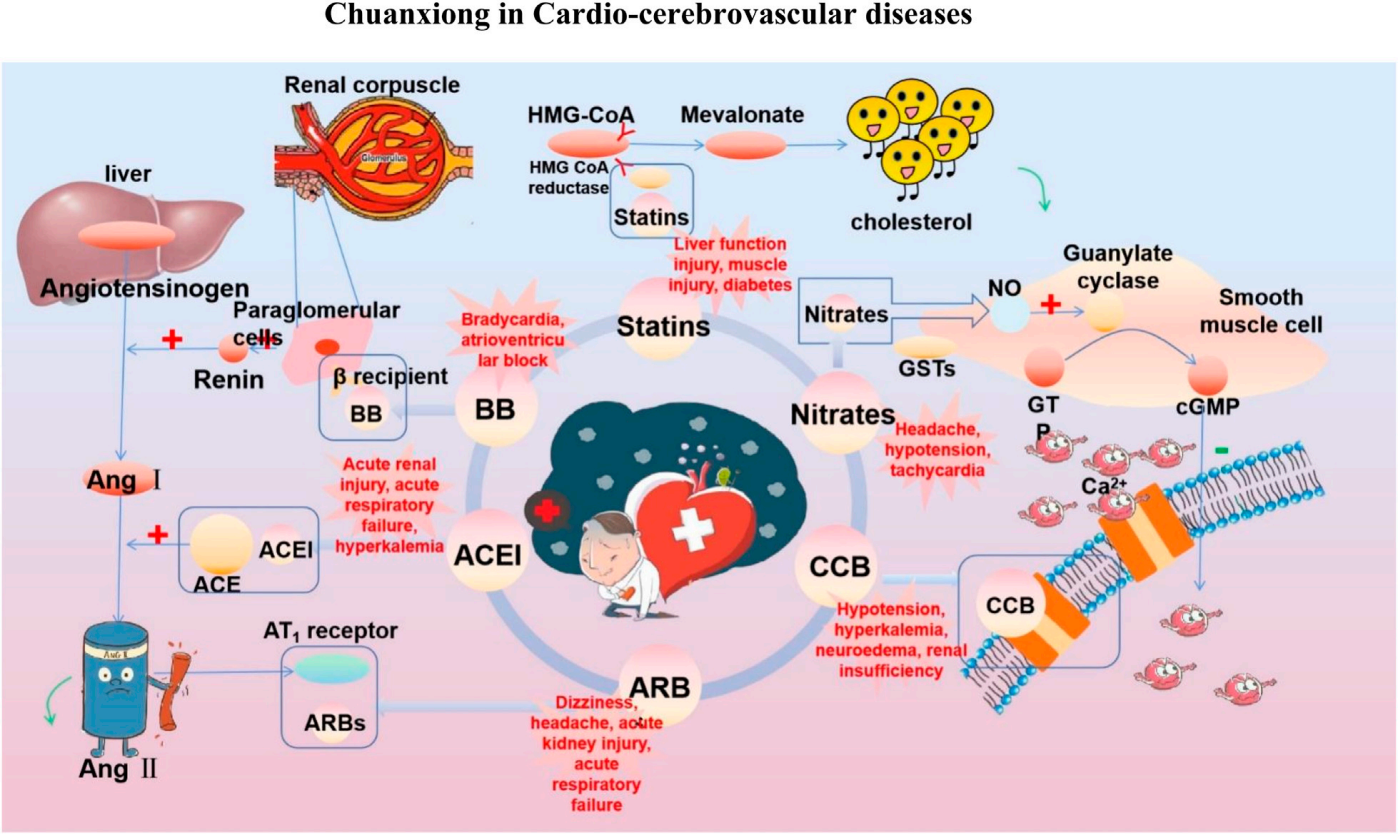
Commonly used drugs for CVD and their mechanisms (the green symbol indicates the effect after administration, and the red symbol indicates the pathological condition. Commonly used drugs for CVDs include statins, BB, ACEI, ARBs, CCB, and nitrates. Angiotensin produced by the liver is converted to Ang I under the action of renin secreted by juxtaglomerular cells, and then to Ang II under the action of ACE. Ang II can bind to AT1 receptor and produce vasoconstriction. BB can bind to β receptor of adjacent glomerular cells and reduce the secretion of renin. ACEI can inhibit ACE and reduce Ang II. ARBs can bind to AT1 receptor and competitively inhibit the binding of Ang II to AT1 receptor. BB, ACEI, and ARBs play a role in the renin–angiotensin system, thereby inhibiting vasoconstriction, reducing blood volume, and lowering blood pressure. CCB can directly block the Ca^2+^ channel on the endothelial cell membrane and reduce Ca^2+^ influx, thereby reducing blood pressure and myocardial contractility. HMG-CoA is transformed into mevalonate by HMG-CoA reductase, and then into cholesterol. Statins can significantly reduce blood cholesterol and LDL by inhibiting HMG-CoA reductase, thus achieving the effect of lowering blood lipid. Nitrates catalyzed the release of NO in the smooth muscle cells by GSTs, and NO activates guanylate cyclase, increasing the content of intracellular second messenger cGMP, and then activating cGMP-dependent protein kinase, which reduces intracellular Ca^2+^ release and extracellular Ca^2+^ influx and relaxes smooth muscle cells).

At present, the clinical treatment of CVDs still mainly uses BB, calcium channel blockers (CCB), ACEI, ARBs, statins, nitrates, etc (Liu et al., 2019b). In a survey of patients with CHD in 24 European countries, the European Heart Association found that of the cardioprotective drugs, BB accounted for 82.6%, ACEI/ARBs for 75.1%, and statins for 85.7% (Kornelia et al., 2016). Statin is the first ideal choice to treat hypercholesterolemia. In addition, drugs such as ACEI, ARBs, BB, CCB, and nitrate are also commonly used for CVDs. However, while it plays a therapeutic role in several ways, it affects normal cells, proteins, and factors, resulting in various toxic effects and side effects (Figure 3); for example, nearly one-third of statin users generally have side effects depending on the dose, and the risk of myopathy is higher at higher doses (Li et al., 2015a).

CAM has gained popularity among health professionals over the past few years because of its preventive mechanisms against the side effects of chemotherapy drugs. On the basis of several acute toxicology studies, CX was found to be highly safe in both gavage experiments and intraperitoneal injection. In the CX gavage experiment, mice's LD_50_ is 7.26 g/kg, or 1460 times the maximum clinical dose of CX. In the CX intraperitoneal injection, mice's LD_50_ is 2.52 g/kg, which is equivalent to 5,091 times of the maximum clinical dose of CX (Ran, 2012). Xia et al. (2018) found that under the 16-fold clinical dose, the decoction of CX may increase mouse fetal stillbirth and absorption fetus, cause sternum and limb bone deformities, and have weak embryotoxicity. Zhang (2014) measured that the LD50 of mice was 1594.92 mg/kg after a single intragastric administration of CX volatile oil. In addition, CX has few adverse reactions in clinical application, mainly facial flushing and emotional agitation, which can improve without additional intervention (Pan, 2012). It is demonstrated that CX is highly safe at normal clinical doses.

The above results are mainly based on the preclinical and clinical research of CX. In life, CX is often boiled together with beef and mutton as health food (Donkor et al., 2016). In Yunnan, China, the tender stems and leaves of CX are often eaten as vegetables (Yang et al., 2013). The characteristics of high safety of CX were explained from the medicinal and edible perspective. However, although natural drugs are generally considered to be safe and non-toxic, they have the characteristics of “multi-links and multi-targets,” they inevitably cause us to think about whether they will produce unknown or uncontrollable effects, and they affect the uptake and transport of drugs and then cause damage. Therefore, as a “homology medicine and food” drug, when evaluating the safety of CX, we must pay attention to being rigorous and objective. We cannot simply identify its high safety. We should study the potential safety hazards from the whole and in part.

## 8 Summary and Prospect

As a natural medicine, CX has less toxic and side effects than other artificial drugs. It has rich effective ingredients, has extensive pharmacological effects, and can be used in many parts, with good quality and at low price. Based on these characteristics, the rhizomes, leaves, and fibrous roots of CX can be used as food and medicine. However, at present, the rhizomes are mostly used and the aboveground parts are abandoned, which not only causes a waste of resources but also makes it difficult to control its quality. At the same time, due to its planting, harvesting, processing, and other links, there are many influencing factors resulting in the differences in its efficacy, including soil fertility, water and nutrients in growth, harvesting method, season, origin, and so on, make its quality differences. Therefore, the establishment of strict quality control and safety testing means can not only strengthen the utilization of resources but also realize its industrialization and internationalization, realize the unified control of CX's quality, and enhance its safety identification. With the development of drug safety research, metabolomics can be used to identify related biomarkers, monitor the overall dynamic changes of metabolites, judge the effect of drugs on the target site, and then realize the scientific prediction of drug safety. In addition, quality markers can also be used to control drug quality to avoid the impact of evaluation based on single component or multiple component indexes on the overall quality monitoring. At present, the safety study of CX is not perfect, so it can be studied by means of metabonomics, equivalent component group discovery technology, serum pharmacochemistry, network pharmacology, quality markers, etc., to provide a basis for safety evaluation and quality control of CX.

Finally, CX has rich pharmacological effects and a wide range of applications. Its basic research is abundant, mainly in the field of CVDs. However, current studies on the direct target and mechanisms of CVDs treatment are still lacking. It requires researchers to carry out more in-depth research from the molecular, cellular, and animal perspectives and provides more comprehensive treatment ideas for CX in the treatment of CVDs through the combination of molecular biology, genomics, metabonomics, and other methods which further promote the progress of human health career. At the same time, in order to optimize the efficacy of CX, studies can also be carried out from the perspectives of preparation and drug delivery way. The preparation of modern new nanometer preparation and the expansion of drug delivery way can improve drug loading, optimize drug delivery efficiency, solve the problems of first-pass effect and blood–brain barrier, achieve more efficient accurate effects, and promote modern CX research from the CVDs point of view.
