# Supplementary figures and images for: Research Advances in Cardio-Cerebrovascular Diseases of Ligusticum chuanxiong Hort
Source: Front Pharmacol. 2022 Jan 31;12:832673. doi: 10.3389/fphar.2021.832673 (PMC8841966; doi:10.3389/fphar.2021.832673)

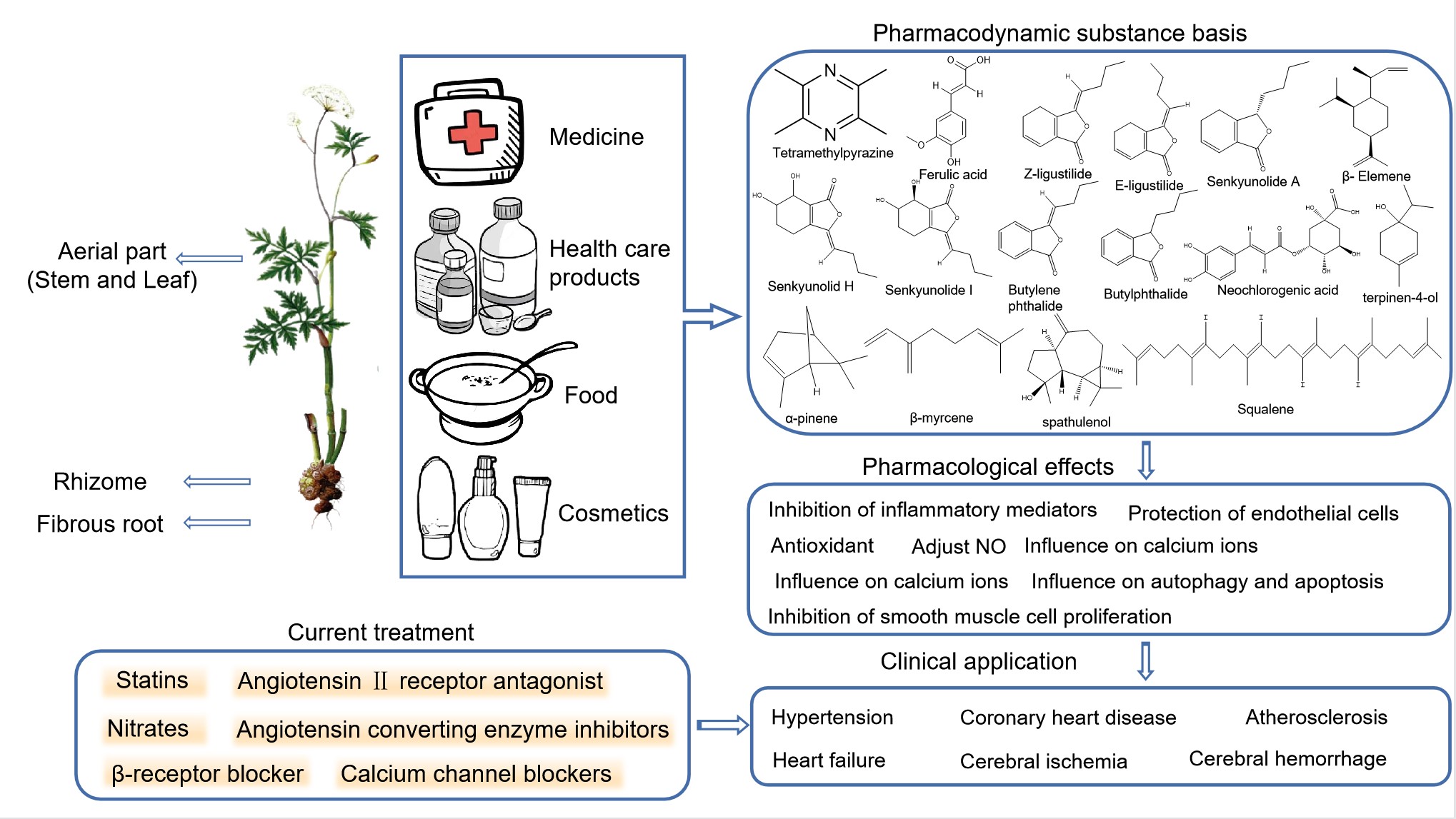

Supplement: Supplementary file 1 [file DataSheet1.zip › Supplementary Material Presentation/Graphical Abstract.jpg]
